# Expanding the Therapeutic Landscape: Sodium-Glucose Co-transporter 2 Inhibitors in Kidney Transplant Recipients

**DOI:** 10.7759/cureus.97193

**Published:** 2025-11-18

**Authors:** Alishba Khan, Muhammad Mohsin Ali, Rizwan Hamer

**Affiliations:** 1 Internal Medicine, University Hospitals Coventry and Warwickshire NHS Trust, Coventry, GBR; 2 Internal Medicine, University Hospitals of Northamptonshire, Kettering, GBR; 3 Nephrology, University Hospitals Coventry and Warwickshire NHS Trust, Coventry, GBR

**Keywords:** chronic renal insufficiency, diabetic nephropathy (dn), kidney transplant recipients, post-transplant diabetes mellitus, post-transplant kidney disease, sodium-glucose co-transporter 2 inhibitors (sglt2i)

## Abstract

Sodium-glucose co-transporter 2 inhibitors (SGLT2i) are well-established oral antidiabetic agents, which also have an important role in slowing the progression of chronic kidney disease and providing cardiovascular protection, particularly in patients with heart failure. The use of SGLT2i in renal transplant recipients is of growing clinical interest; however, the majority of supporting evidence highlighting their efficacy and safety in this population comes from small, retrospective observational studies rather than large randomized trials. This review synthesizes current evidence on the efficacy and safety of SGLT2i in renal transplant recipients, including both diabetic and non-diabetic individuals. Literature search was carried out on multiple databases using a combination of both keywords and free-text search. There is strong evidence from several studies to support a beneficial role of SGLT2i in diabetic renal transplant patients in terms of improving glycaemic control, reducing proteinuria, preserving renal allograft function, and in reducing the risk of major adverse cardiovascular and renal effects and mortality in transplant recipients. There is encouraging but insufficient evidence to support the routine use of SGLT2i in non-diabetic renal transplant recipients, and there is a significant literature gap regarding transplant patients with impaired renal function. SGLT2i appear to have a good safety profile with the predominant side effect being urinary tract infections, which were not significantly associated with allograft dysfunction or loss. Further studies are needed to clarify and validate the cardio-renal and metabolic benefits of SGLT2i in renal transplant recipients that are observed in other patient populations.

## Introduction and background

Chronic kidney disease (CKD) globally affects more than 850 million people, with a significantly higher prevalence compared to other chronic diseases, such as diabetes and cancer [1]. CKD is defined by long-term changes in renal structure, function, or both over a minimum period of three months with health implications. CKD is further categorized based on etiology, presence and degree of albuminuria, and glomerular filtration rate (GFR) [1]. The stages of CKD based on GFR categories range from one to five, with stage five CKD or end-stage renal disease (ESRD) categorized by GFR values <15 ml/min/1.73 m^2^. However, diagnosis of CKD does not solely rely on decreased GFR, and also includes the presence of one or more markers of kidney damage, particularly albuminuria (albumin-creatinine ratio ≥30 mg/g), over a minimum period of at least three months [1]. Other markers of kidney damage include persistent hematuria, urine sediment abnormalities, abnormalities revealed on histology or structural abnormalities detected by imaging, or electrolyte and acid-base abnormalities due to tubular disorders [1].

CKD is generally considered a progressive condition with increasing albuminuria and declining GFR over time, leading to an increased risk of cardiovascular complications, renal failure, and even premature death [2]. The advent of treatments such as renin-angiotensin system (RAS) inhibitors (including angiotensin-converting enzyme inhibitors (ACEis) and angiotensin II receptor blockers (ARBs)), non-steroidal mineralocorticoid receptor antagonists (MRAs), and sodium-glucose co-transporter 2 inhibitors (SGLT2i), which are cardioprotective and renal protective, has changed the landscape of CKD management [1]. 

Renal transplantation is the ultimate preferred treatment for end-stage renal disease, which can prolong and improve the quality of life of these patients. However, renal transplant recipients who are chronically immunosuppressed can still face serious consequences from existing comorbidities such as diabetes; furthermore, 15-30% of non-diabetic patients are also at risk of developing post-transplant diabetes [3,4]. This is associated with an increased risk of early cardiovascular disease, renal graft dysfunction and failure, and can also increase mortality rates in renal transplant recipients [4]. There is an increasingly emerging focus on treatments that can limit these complications and improve long-term graft survival, and this review explores whether SGLT2i are beneficial in limiting complications and improving graft and patient survival in renal transplant recipients.

SGLT2i, historically used to treat hyperglycemia in patients with type 2 diabetes mellitus (T2DM), have been shown to reduce CKD progression and provide cardiovascular benefits in both diabetic and non-diabetic patients across a series of clinical trials [5-7]. While canagliflozin, the first SGLT2i to be approved for CKD treatment in type 2 diabetics, could be initiated at GFR >30 ml/min/1.73 m^2^, both dapagliflozin and empagliflozin are approved for CKD management irrespective of diabetic status and can be initiated at even lower GFR values (≥20 ml/min/1.73 m^2^) [8]. The Kidney Disease: Improving Global Outcomes (KDIGO) guideline update 2024 recommends the use of SGLT2i in diabetic patients with CKD and GFR ≥ 20 ml/min/1.73 m^2^. In all other patients, regardless of diabetic status, SGLT2i are recommended for CKD patients with GFR ≥20 ml/min/1.73 m^2^ who either have a urine albumin-creatinine ratio (ACR) ≥20 mg/mmol, or have heart failure, irrespective of the degree of albuminuria. In patients with GFR 20-45 ml/min/1.73 m^2^, who have albuminuria with a urine ACR <20mg/mmol, the KDIGO guidelines suggest using SGLT2i to reduce the risk of renal failure, with a 2b level of evidence [1,9]. 

The role of SGLT2i in managing CKD is well-founded, with a large meta-analysis of 13 trials with over 90,000 adult patients, conducted by the Nuffield Department of Population Health Renal Studies Group and the SGLT2 inhibitor Meta-Analysis Cardio-Renal Trialists' Consortium (2022), demonstrating evidence of reduction in renal disease progression, risk of AKI, and risk of major adverse cardiovascular events (MACE) [10]. Both diabetic and non-diabetic patients were shown to have a reduced risk of renal disease progression with SGLT2i initiation compared to placebo, with up to 37% risk reduction; similarly, the risk of acute kidney injury, hospitalization due to heart failure, and risk of cardiovascular death also reduced significantly in both diabetic and non-diabetic patients who were allocated to SGLT2i. The absolute benefits of SGLT2i outweighed any potential serious side effects, such as ketoacidosis or risk of limb amputation [10]. SGLT2i may lead to an initial decline in GFR after initiation owing to their renal mechanism of action; however, this does not indicate CKD progression and does not mitigate the long-term beneficial cardiovascular and renal effects of these drugs [11,12]. 

A caveat of the KDIGO guidelines is that the recommendations for initiating SGLT2i were based on pivotal clinical trials, which excluded patients who had undergone renal transplantation due to concerns regarding infection risks and immunosuppression interaction, leading to under-evaluation of the safety and efficacy of SGLT2i in these patients and creating an evidence gap [10]. Consequently, the current KDIGO guideline update does not make recommendations regarding the use of SGLT2i in patients with renal transplants.

Certain factors associated with SGLT2i use, such as an increase in urinary tract infections and risk of renal graft dysfunction, may limit their use in post-renal transplant patients [13]. Recently, some case series and observational studies have focused on this patient cohort, both with and without diabetes, in order to determine a definitive role for SGLT2i in renal transplant recipients; however, these data are from small-scale studies rather than large randomized trials. The Renal Lifecycle Trial, a multicenter, randomized, placebo-controlled trial, is also underway to focus on the role of SGLT2i in subgroups of patients who were excluded from earlier trials, including renal transplant recipients with GFR ≤45 ml/min/1.73 m^2^ [14].

With the widespread use of SGLT2i in both diabetic and non-diabetic chronic kidney disease patients, it is pertinent to explore whether their role can be expanded to include renal transplant recipients as well. The objective of this narrative review is to consolidate and present available data on the role of SGLT2i in renal transplant recipients. It also aims to provide a framework for future research studies as well as clinical guidelines. 

## Review

Methods

Literature search was carried out using both open text as well as keyword search. Keywords included ‘SGLT2i,’ ‘chronic kidney disease,’ ‘renal transplant,’ ‘kidney transplant,’ ‘live-donor transplant,’ ‘cadaveric transplant,’ ‘diabetes mellitus,’ ‘dapagliflozin,’ ‘empagliflozin,’ and ‘canagliflozin,’ which were combined using Boolean variables. Free-text search was also carried out to enhance data availability for the topic under consideration. Search limitations were set for studies published after 2010. Various databases were searched, including PubMed, Ovid MEDLINE, Embase, and Google Scholar, and the results were then collated to remove duplicates and choose articles for final review. Case reports, case series, retrospective and prospective observational studies, randomized controlled trials (RCTs), non-randomized trials, and systematic reviews and meta-analyses published in English, which included only human adult subjects and had full text available were included for final review. Narrative reviews, commentary articles, and letters to editor were excluded from the final review. Due to the narrative nature of the current review, a formal risk of bias assessment was not carried out; furthermore, quantitative analysis was not done due to the heterogeneity of the included studies.

Discussion

Several studies have shown a beneficial effect of SGLT2i in renal transplant recipients with a good safety profile. These beneficial effects include improved diabetic control as evidenced by reduction in glycated hemoglobin (HbA1C) levels, improvement in blood pressure control, weight reduction, and stability of renal graft function with reduced incidence of acute kidney injury [15]. These beneficial effects and safety profile of SGLT2i in diabetic and non-diabetic renal transplant recipients are discussed below.

Diabetes in Post-renal Transplant Patients

Renal transplant patients can face both short- and long-term adverse effects due to diabetes. Post-transplant diabetic patients often have diabetes prior to transplant, but can also develop new-onset diabetes after transplant (NODAT), also known as post-transplant diabetes mellitus (PTDM) [16]. Longstanding diabetes can lead to morphological changes in the glomerular architecture, eventually leading to mesangial expansion and nodular infiltration of the mesangial matrix and glomerulosclerosis. These changes clinically manifest as a reduction in GFR, an increase in urinary albumin excretion, or both, hallmarking diabetic kidney disease (DKD) [17,18].

PTDM is clinically distinct from type 1 and type 2 diabetes, with multifactorial pathogenesis associated with risk factors, such as obesity, viral infections (hepatitis C and cytomegalovirus), acute transplant rejection, and chronic immunosuppressant use (particularly calcineurin inhibitors and steroids) [18]. PTDM mostly occurs in the first year after renal transplant and requires oral glucose tolerance test (OGTT) as the gold standard diagnostic method, which can either be performed two months after transplant or after the patient has clinically stabilized and is established on immunosuppressive regimen [19,20]. PTDM has been associated with increased risk of renal graft failure and can lead to a significantly increased risk of all-cause mortality, compared to non-diabetic patients [20,21].

Preventing PTDM is important, especially in higher-risk patients, such as those with hypertension, hypertriglyceridemia, impaired fasting glucose, metabolic syndrome, hepatitis C virus (HCV) infection, or family history of diabetes [18,22]. Preventative techniques include weight reduction, lipid management, lifestyle modifications, and physical activity, as well as medication adjustments, such as using an immunosuppressant regimen without glucocorticoids or using azathioprine or mycophenolate to reduce the dosage of calcineurin inhibitors [23,24].

Medical management of PTDM can be challenging, not only due to the potential adverse effects of antidiabetic drugs on renal function, but also because efficacy and safety of certain common antidiabetic therapies still need to be established in renal transplant patients [18]. An international consensus meeting on management of PTDM recommended the use of early exogenous insulin administration for treatment of postoperative hyperglycemia after transplantation to prevent PTDM [25]. A large multicenter RCT on the role of basal insulin therapy versus standard of care in PTDM prevention in patients with post-transplant hyperglycemia did not find a significant difference in incidence of PTDM at 12 months in both groups (12.2% vs 14.7%); however, after per-protocol analysis was adjusted for polycystic kidney disease and glomerular nephritis, a significant difference at one-year endpoint (PTDM) development was observed [26].

The role of other antidiabetic drugs in the management of PTDM is also currently being investigated. A pilot-randomized trial found metformin to be safe and tolerable in renal transplant patients with impaired glucose tolerance; however, it did not significantly improve glycemic parameters, indicating more robust trials are necessary to establish definitive metabolic benefits in this population [27]. A large retrospective analysis showed that metformin use in kidney transplant recipients on tacrolimus may reduce T-cell-mediated rejection and graft failure risk without increasing adverse outcomes, supporting a potentially protective immunomodulatory role that warrants further prospective investigation [28].

The role of dipeptidyl peptidase 4 inhibitors (DPP4i) is also under investigation for PTDM management: a small cross-over randomized study with nineteen renal transplant patients who developed NODAT showed that sitagliptin increased insulin secretion and also decreased both fasting and post-prandial glucose levels in these patients, with a good short-term safety profile over the four-week duration of the study [29]. In a slightly larger, single-center randomized controlled study, sitagliptin was associated with improved two-hour OGTT values at three and six months compared to placebo, and also led to an absolute risk reduction of 18.06% in the incidence of new-onset PTDM at three months; however, these findings were not statistically significant [30].

Another class of drugs with an emerging and promising role in both T2DM and PTDM is glucagon-like peptide-1 receptor agonists (GLP1RA). Combining GLP1RA with insulin in PTDM management after solid organ transplantation has been shown to achieve a similar HbA1C target goal after a one-year period as insulin alone, with the added benefit of decreased insulin use by 69% and fewer insulin injections per week [31]. A meta-analysis of 338 kidney transplant recipients with either T2DM or PTDM showed that the use of GLP1RAs led to a decrease in proteinuria, improved glycemic control and facilitated weight loss without impacting immunosuppressant (tacrolimus) levels and without serious adverse effects [32].

Role of SGLT2i in Post-transplant Diabetic Patients

Several research studies, mainly observational and retrospective studies, have demonstrated a significantly positive impact with the use of SGLT2i in diabetic renal transplant recipients. However, SGLT2i are not currently recommended in the diabetic renal transplant population as per the KDIGO guidelines, as patients on intensive immunosuppressant therapy after solid organ transplants were excluded from the pivotal CKD trials that led to the initial approval of SGLT2i use [8]. The current evidence for SGLT2i use in post-transplant diabetic patients is summarized below.

A retrospective analysis of eight diabetic patients (two with pre-existing T2DM, six with PTDM) who had undergone renal transplant and had stable renal function with CKD stage I or II (estimated GFR (eGFR) >60) showed that SGLT2i use improved glycemic control with reduction in HbA1c levels, and also reduced body mass index (BMI) after a period of 12 months, without any significant deterioration in renal function or risk of recurrent urinary tract infections (UTIs) [13]. Similar findings were reported in a case series of ten renal transplant patients (40% having PTDM) with stable allograft function, in whom adding Empagliflozin to pre-existing antidiabetic medications led to decrease in median HbA1c levels at 12 months, and also decreased insulin requirement by 10-25% without any adverse effect on allograft function (demonstrated by stable eGFR over 12 month period) [33].

Findings from retrospective studies vary in regard to the impact of SGLT2i on glycemic control in diabetic renal transplant patients. In a retrospective single-center analysis of 50 patients (80% with pre-existing diabetes), SGLT2i initiation was found to be safe and led to significant weight loss without any major impact on renal function; however, it had no significant impact on glycemic control or insulin requirements [34]. Similarly, a retrospective inverse probability of treatment weighting analysis of 85 renal transplant patients with pre-existing diabetes showed no significant impact of SGLT2i initiation on changes in HbA1c levels [35]. However, another retrospective analysis of 39 diabetic patients (44% with PTDM) who were initiated on SGLT2i showed significant improvement in HbA1c levels at both three and 12 months compared to baseline, without any significant impact on renal function or tacrolimus exposure [36]. A retrospective study comparing the efficacy and safety of SGLT2i and GLP-1 receptor agonists in diabetic renal transplant recipients showed significant reductions in HbA1c levels, BMI, and albuminuria in patients treated with SGLT2i compared to the control group, with minimal adverse effects and a comparable safety profile [37].

In a prospective, interventional pilot study on the role of empagliflozin in renal transplant recipients, a small sample of patients (n=14) diagnosed with PTDM with established insulin therapy were started on empagliflozin once daily while insulin was washed out over a three-day period. These patients were followed up with OGTT at baseline, four weeks, and 12 months. Empagliflozin was found to be clinically inferior when used as monotherapy in patients on prior insulin treatment after four weeks of the study. After 12 months, with some patients having reintroduced insulin for PTDM management, empagliflozin did not significantly improve glycemic control but did lead to significant reductions in body weight, waist circumference, and BMI [38]. Another prospective pilot study examining the role of canagliflozin in stable renal transplant patients with pre-existing diabetes or NODAT showed significant improvement in blood pressure and HbA1c, as well as a reduction in body weight after six months of treatment, without any significant allograft dysfunction (based on rise in serum creatinine levels) or drug-related adverse effects [39].

A multicenter comparison of diabetic renal transplant recipients who received SGLT2i (n=36) versus those who did not (n=21) showed significant proteinuria reduction in the SGLT2i group, without adverse effects on allograft function or increased risk of genital infections and UTIs [40]. In renal transplant recipients with PTDM and stable renal function and immunosuppressant regimen, a prospective, double-blind and randomized study involving 44 patients compared the efficacy of once daily empagliflozin versus placebo, and showed a significant reduction in HbA1c levels (-0.2% vs 0.1%) and body weight (two and a half kg reduction versus one kg mean increase in body weight in placebo group) without any significant impact on allograft function or immunosuppressant levels [41].

The efficacy of SGLT2i in diabetic renal transplant patients has also been highlighted by larger multicenter studies. A large observational multicenter study of 339 diabetic renal transplant patients showed that over six months, SGLT2i led to a significant reduction in body weight, systolic and diastolic blood pressure, HbA1c and fasting glucose levels, as well as serum uric acid levels and insulin usage. These benefits of SGLT2i were independent of the type of diabetes (pre-existing versus PTDM) or the type of SGLT2i used and appeared to be maintained over an extended follow-up period of 12 months [42]. Another large multicenter cohort of over 2000 diabetic renal transplant patients in Korea was analyzed for the impact of SGLT2i after more than three months of use on the composite outcome of all-cause mortality, death-censored graft failure (DCGF), and serum creatinine doubling. Compared to the control group, the SGLT2i group of 226 patients had a significantly lower risk of the composite outcome in propensity score-matched models, with a persistent reduced risk of DCGF on multivariate analysis. A minor caveat of SGLT2i use was an acute dip of ≥10% in the eGFR in the first month, observed in 15.6% of SGLT2i users; however, this recovered thereafter without persistent eGFR decline or allograft dysfunction [43]. A large-scale study of diabetic renal transplant patients over an eight-year period from the TriNetX database compared new users of SGLT2i and DPP4i for long-term outcomes. In the propensity score-matched analysis, SGLT2i users were found to have a lower risk of dialysis initiation (hazard ratio (HR) 0.694) and all-cause mortality (HR 0.687) compared to DPP4i users, without any significant differences in risk of graft rejection, hospitalization, or post-transplant infections [44].

The cardiovascular benefits of SGLT2i are well known, marking their important and multifactorial cardioprotective role in both diabetic and non-diabetic patients with heart failure [45]. These cardioprotective effects have also been shown in renal transplant patients. An analysis of 750 diabetic renal transplant recipients, of which 129 received SGLT2i, showed a significant reduction in the incidence of major adverse cardiovascular events (MACE) in patients receiving SGLT2i (4.7% vs 12.6%, p=0.015). Analysis for various MACE components showed a significant reduction in the incidence of myocardial infarction (MI) and death from cardiovascular causes in the SGLT2i group; however, the incidence of hospitalization for heart failure and risk of stroke did not differ significantly between the two groups. These protective effects of SGLT2i on MACE were consistent in the propensity-matched analysis after accounting for confounding factors; the cardioprotective benefits were more evident in patients aged <60 years who had a higher BMI and well-controlled glucose levels [46].

In another comprehensive analysis of over fifty thousand diabetic renal transplant recipients from the TriNetX database, 1970 SGLT2i users (in the first three months post-transplant) were propensity-matched to non-users and compared to determine reductions in MACE, all-cause mortality, and major adverse kidney events (MAKE). At a median follow-up period of 3.4 years, SGLT2i were found to be associated with significantly reduced mortality rate (2.08% vs 9.54%; adjusted hazard ratio (aHR) 0.32); MACE incidence (4.44% vs 13.87%; aHR 0.52); and MAKE incidence (8.93% vs 22.54%; aHR 0.52). Subgroup analysis showed that these effects in SGLT2i were more pronounced in those concurrently using steroids and tacrolimus; the reduction in mortality and MAKE incidence was also more evident in patients without proteinuria, while the reduction in MAKE incidence was more pronounced in diabetic patients on insulin and in patients without heart failure. While the frequency of acute kidney injury and incident dialysis after renal transplant was lower in the SGLT2i group, stroke and acute MI incidence did not vary significantly between SGLT2i users and non-users [4]. Both of these major studies exploring the cardiovascular benefits of SGLT2i did not stratify their findings by type of diabetes (pre-transplant vs PTDM).

Role of SGLT2i in Post-transplant Non-diabetic Patients

Proteinuria is both a cardiovascular and renal risk factor, leading to loss of renal function and renal disease progression in both diabetic and non-diabetic patients [47]. Persistent and worsening proteinuria after renal transplantation indicates allograft dysfunction and can be associated with poor outcomes, such as increased mortality risk, increased MACE incidence, and graft loss [48]. The anti-proteinuric role of SGLT2i has been shown to delay the progression of renal disease, even in patients with nephrotic-range proteinuria [49]. Reducing proteinuria in the post-renal transplant population could therefore translate into improving graft outcomes. Based on this hypothesis, a small observational study of 22 non-diabetic renal transplant recipients demonstrated significant proteinuria reduction (1.68 to 1.06 g/g, p=0.025) after six months without significant adverse effects. The use of SGLT2i also led to a mild but significant reduction in eGFR, which was more pronounced in patients with chronic rejection or recurrent underlying renal disease [50].

A novel surrogate endpoint for various stages of renal disease progression is eGFR slope, with a reduction in eGFR slope denoting slower renal disease progression and lower risk of renal failure leading to renal replacement therapy [51]. In a recently published single-center cohort of both diabetic and non-diabetic renal transplant recipients, SGLT2i led to a significant annual eGFR slope reduction of 1.2 ml/min/1.73 m^2^ and caused a significant reduction in the urine protein-creatinine ratio (UPCR) at six months, demonstrating a slower rate of decline in renal function. These effects were independent of diabetic status, thereby highlighting the potential renoprotective role of SGLT2i for non-diabetic renal transplant patients [52].

Safety Profile for SGLT2i in Post-transplant Patients

The adverse effects of SGLT2i are related to their renal mechanism of action. The induction of glycosuria can lead to bacterial and fungal overgrowth, manifesting as UTIs and genital mycotic infections, and the diuretic effect of these drugs can deplete intravascular volume, thereby increasing the risk of acute renal injury [53]. Additional concerns regarding their use in diabetic patients include the risk of diabetic ketoacidosis, especially euglycemic ketoacidosis [53]. In renal transplant recipients, safety concerns regarding SGLT2i also include their effect on allograft function and survival, and the risk of severe infections and Fournier's gangrene in immunosuppressed patients [54].

However, several studies on the role of SGLT2i in both diabetic and non-diabetic renal transplant recipients have noted an overall favorable safety profile with minimal serious adverse effects. A large observational multicenter study by Fructuoso et al. (2023) examined the adverse effects of SGLT2i after six months of treatment in diabetic renal transplant recipients as the primary outcome. Their findings revealed UTIs as the predominant adverse effect of SGLT2i (13.6%), which were more common in women and were not associated with the type of SGLT2i used or with the type of diabetes (pre-existing or PTDM). UTI subgroup analysis with extended follow-up of one year showed similar incident rates in diabetic renal transplant recipients and a reference group of non-diabetic renal transplant recipients [42]. A small proportion of patients in their study had AKI over a one-year follow-up period (1.8%), with AKI prevalence higher in patients with baseline eGFR <40 ml/min/1.73 m^2^. There were no acute graft rejection cases, and one case of graft loss was attributed to SGLT2i due to recurrent urinary candidiasis and fungal pyelonephritis [42].

Studies have not shown a significantly increased risk of genitourinary infections with SGLT2i use in renal transplant recipients. Analysis of a large database of renal transplant recipients with propensity-score-matched groups of SGLT2i users and non-users revealed no significant risk increase for UTIs (aHR 1.02) or urinary candidiasis (aHR 1.30) with SGLT2i use [4]. Similar findings were reported in another study with propensity-score-matched SGLT2i users and non-users groups: there was no difference in the risk of all UTIs and bacterial UTIs with SGLT2i, and while fungal UTIs occurred more frequently in the SGLT2i group, their incidence was quite rare (0.09 events/100 patient years) [46]. The studies referenced above also did not find any incidences of euglycemic ketoacidosis in their studied populations. Table 1 shows the reported adverse effects and safety outcomes with SGLT2i use in renal transplant patients from observational and randomized studies.

**Table 1:** Adverse effects and safety impact of SGLT2i in renal transplant recipients. AKI: acute kidney injury; UTI: urinary tract infection; SGLT2i: sodium-glucose co-transporter 2 inhibitors.

Authors and year	Type of SGLT2i and number of patients on SGLT2i (n)	Urinary tract infections	Genital mycosis	Diabetic or euglycemic ketoacidosis	Acute kidney injury	Acute graft rejection or graft loss	Other adverse effects	Safety impact on patients
Halden et al. 2019 [41]	Empagliflozin (24)	3 (12.5%)	1 (4.2%)	0	0	0	Facial swelling, genital itching, and hematuria (each 4.2%)	One patient withdrawn due to urosepsis
Schwaiger et al. 2019 [38]	Empagliflozin (14)	3 (21%)	0	0	Not recorded	Not recorded	One case of mild hyponatremia, one case of balanitis	One hospitalization due to pneumonia
Shah et al. 2019 [39]	Canagliflozin (24)	0	0	0	0	0	None	One patient dropped out after two weeks due to creatinine rise
Song et al. 2020 [34]	Empagliflozin, canagliflozin, and dapagliflozin (50)	7 (14%)	1 (2%)	0	0	0	None	Five patients discontinued due to UTIs, one discontinued due to genital yeast infection
Hisadome et al. 2021 [35]	Canagliflozin, ipragliflozin, luseogliflozin, empagliflozin, dapagliflozin, and tofogliflozin (28)	2 (6.4%)	0	0	Not recorded	1 (2.5%)	One patient had other adverse effects, but not specified within the study	Biopsy-proven acute graft rejection within 48 weeks in one patient
Lim et al. 2022 [43]	Empagliflozin or dapagliflozin (226)	11 (4.9%)	1 (0.4%)	0	0	0	None	None
Lemke et al. 2022 [36]	Empagliflozin, canagliflozin, and dapagliflozin (39)	6 (15.4%)	0	1 (2.6%)	2 (5.1%) – attributed to simultaneous UTI and acute heart failure exacerbation rather than SGLT2i-related renal compromise	0	2 (5.1%) patients with diabetic foot ulcers 2 (5.1%) episodes of mild hypoglycemia	Four patients discontinued due to declining kidney function. Three discontinued due to infections (one fungal pneumonia, one urinary sepsis, one recurrent UTIs with pyelonephritis), one patient discontinued due to diabetic ulcer and non-healing wound, one discontinued due to hypoglycemia
Sánchez Fructuoso et al. 2022 [42]	Canagliflozin, dapagliflozin, empagliflozin, or ertugliflozin (339)	48 (14%) over 12-month follow-up period	5 (1.5%)	0	6 (1.8%)	One (recurrent urinary infections leading to fungal pyelonephritis)	Polyuria (16, 4.7%); hypoglycemia (4, 1.2%); diarrhea and weight loss (2, 0.6% each)	Drug was suspended definitively in 34 patients (10 due to UTI, five due to AKI, three due to genital mycoses, and the rest for other causes)
Demir et al. 2023 [40]	Empagliflozin or dapagliflozin (36)	6 (16.7%)	0	0	0	Four acute graft rejection episodes, only one occurred after SGLT2i use	None	One case of urosepsis requiring hospitalization. Overall, 3 (8.3%) hospitalized
Mahmoud et al. 2023 [37]	Canagliflozin (98)	15 (15.3%)	1 (1.0%)	0	0	Not recorded	None	Five patients had recurrent UTIs, and one developed septic shock requiring hospitalization. No drug discontinuation recorded
Lim et al. 2024 [46]	Empagliflozin or dapagliflozin (127)	12 (9.4%)	0	0	Not recorded	Not recorded	None	None

Directions for Future Research

This review highlights an important and multifaceted role of SGLT2i in both diabetic and non-diabetic renal transplant patients. Nevertheless, there are several avenues for future research to explore. Prospective RCTs are required to confirm the cardio-renal protective role of SGLT2i, which has been highlighted by observational studies.

The majority of observational studies concerning SGLT2i have included patients with stable renal disease, usually with an eGFR ≥45 ml/min/1.73 m^2^. An ongoing large-scale randomized multicentric trial, the Renal Lifecycle trial, aims to explore the role of SGLT2i (dapagliflozin once daily) in patients with severe CKD, including both diabetic and non-diabetic patients who have undergone renal transplant and have an eGFR ≤45 mL/min/1.73 m^2^ at least six months after transplantation [14].

Several other ongoing trials are exploring various facets of SGLT2i inhibition in renal transplant patients. For instance, the recently completed but yet unpublished INFINITI2019 trial primarily aimed to determine a beneficial impact of dapagliflozin in reducing blood pressure in renal transplant recipients (both with or without pre-existing diabetes or PTDM), in order to elucidate the potential cardio-renal protective role of these drugs [55]. The DEAKTransplant trial aims to explore the role of SGLT2i in preserving renal function, reducing renal interstitial fibrosis, and improving metabolic risk factors for graft failure, such as visceral obesity, raised blood pressure, and glucose intolerance [56].

Several other trials are prospectively exploring the role of SGLT2i on allograft dysfunction and loss [57], as well as the interaction of SGLT2i with other novel antidiabetic drugs, such as GLP1RAs, in exerting a synergistic effect on improving renal transplant outcomes [58]. In non-diabetic renal transplant recipients, no long-term data on cardioprotective effects, mortality, or allograft function are yet available. The SGL-TX-GFR clinical trial is a phase 4 double-blind, randomized, placebo-controlled trial, which is currently underway to determine whether 18 months of daily SGLT2i use can preserve renal function as an add-on to standards of care in non-diabetic renal transplant recipients: data from this clinical trial will be instrumental in highlighting the future role of SGLT2i in this patient population [59]. 

Prospective trials should also take into account non-diabetic populations, such as patients with heart failure, who can benefit from the cardioprotective effects of SGLT2i. It would also be useful to determine the ideal timeline of initiation of SGLT2i after transplant, and to determine if an acute reduction in eGFR after SGLT2i initiation has any impact on long-term graft and patient survival [60]. Moreover, considering the side effects of SGLT2i, such as recurrent genitourinary tract infections, prospective studies should also consider their impact on the quality of life of renal transplant recipients. Future studies can also explore long-term cost-effectiveness and the impact of using SGLT2i on future hospitalization events in renal transplant recipients.

## Conclusions

Current and emerging evidence supports the multifaceted role of SGLT2i in both diabetic and non-diabetic renal transplant patients. For diabetic patients, SGLT2i have shown improvements in glycemic control, reduction in insulin usage, and preservation of renal function, as well as cardiovascular benefits with reduced major adverse cardiac events and all-cause mortality. While the evidence of their use in non-diabetic patients is limited, future research, including ongoing studies, will be helpful in establishing a guideline-based approach to early SGLT2i initiation. SGLT2i are overall safe and efficacious and will be a useful addition to the current standard of care in managing renal transplant recipients. Clinicians should, nevertheless, exercise judicious decision-making before initiating these drugs. Multi-disciplinary discussion should be done before considering these medications in renal transplant recipients, and close monitoring should be performed, particularly in patients with pre-existing renal dysfunction and those at high risk of allograft dysfunction or failure. 
